# Mechanical Property Test of Grass Carp Skin Material Based on the Digital Image Correlation Method

**DOI:** 10.3390/s22218364

**Published:** 2022-10-31

**Authors:** Mei Zhang, Pengxiang Ge, Zhongnan Fu, Xizuo Dan, Guihua Li

**Affiliations:** 1 School of Electrical Engineering and Automation, Anhui University, Hefei 230601, China; 2 School of Instrument Science and Opto-Electrics Engineering, Hefei University of Technology, Hefei 230009, China

**Keywords:** grass fish skin, digital image correlation, mechanical characteristics, measurement

## Abstract

Fish is a common and widely distributed creature. Its skin has a unique physiological structure and plays an important role in many fields. Fish skin also has important potential value for bionics research. This study aims to provide a method and a reliable data for the study of bionics. A method of measuring the mechanical properties of fish skin samples using a binocular stereo digital image correlation (DIC) system combined with a synchronous tensile testing machine was proposed. The mechanical properties (e.g., elastic modulus *E* and strain) of grass fish skin samples (GFSA) were tested in hydrophilic and dry states. A dual-frequency laser interferometer was used to calibrate the tensile testing machine synchronously, and the feasibility and strain accuracy of DIC in GFSA measurement were verified by finite element method (FEM). The results show differences in the mechanical properties of GFSA between different individuals, different parts, and different states. Under the same stress, the head was easy to deform, and the strain was the largest, and *E* was the smallest. The tail result was the opposite of the head result.

## 1. Introduction

Fish is a rich natural resource with a wide range of application values in many fields such as life, biology, medicine, and bionics [1,2,3,4,5]. Research on fish skin is mainly focused in the biological and medical fields, or its application in biomimetic materials. Few study methods are available for reference on the biomechanical characteristics of fish skin, and effective citation data resources are lacking. In traditional biomaterial-related research, extensometers or strain gauges are usually used to analyze the relevant properties of materials by means of tensile or pressure tests. However, the two traditional methods are single-point measurements and do not meet the current and future needs for full field measurement. At the same time, fish skin is a deformable material. Operating the physical measuring gauge also can easily interfere with the surface of the material, which has a direct impact on the measurement accuracy. Therefore, this study aims to introduce an advanced noncontact measurement technology to test and study the biomechanical characteristics of grass carp skin.

Studies related to fish skin, which is an important part of fish body tissue, have attracted the attention of relevant researchers. In the medical field, Dured [2] used fish skin to treat necrotizing dermatitis in diabetic patients. Sang J L [4] created a polymer material to coat medical devices according to the smoothness of the loach surface, which reduced the biological pollution of medical equipment. Ge [5] studied fish genome to expand the genome database and provide data support for new marine drugs. Szewciw et al. [3] investigated the structure and mechanical properties of striped bass skin through tensile and puncture experiments combined with optical microscopy. Franck J V et al. [6] explored the excellent protective properties of fish skin and showed that fish skin has various strain hardening and stabilization mechanisms, which effectively protect itself. Shark skin has the function of reducing drag, and Kim H T [7] used optical reconstruction technology to fabricate biomimetic shark skin, which can be effectively used on low-drag surfaces. Therefore, fish skin has great research value.

Digital image correlation (DIC) has rich practical experience in material deformation measurement [8,9,10,11] because of its characteristics of full field, noncontact, and wide application range [12,13,14]. In recent years, DIC has begun to emerge in biomaterial-related research, and it has shown good performance in biological soft and hard tissue analysis. Palanca [15] comprehensively reviewed the research development of DIC in biomechanics in 2015. In the field of clinical medicine, Morosato [16] used a binocular DIC system to measure the translation and rotation of the external displacement body in the study of the hip acetabular, which is important to improve the success rate of transplantation. Alice [8] used DIC to measure the elastic modulus of skeletal tissue and verified the related results of DIC by four-extensometer technique. Chen Y F [17] combined an optical parameter measurement system with DIC to analyze the relationship between biological tissue strain changes and optical parameters. Thus, when the Young’s modulus of biological tissue is known, the relative optical parameters such as stress factor can be characterized. Fish skin has a unique natural texture, and Petr C [18] used machine vision technology to estimate fish population changes based on the dotted pattern of fish skin.

In view of the wide application of DIC in the research of biomaterials, this study applies DIC technology to the mechanical properties test of grass carp skin, analyzes the mechanical properties of grass fish skin samples (GFSA), and conducts exploratory research for related material field, which provides important data support for bionics research. Uniaxial tensile stress was applied to GFSA by manipulating a high-precision tensile testing machine. A dual-frequency laser interferometer simultaneously calibrated the displacement accuracy of the testing machine during the stretching process. The binocular stereo DIC system captured speckle images during the entire deformation process of grass carp skin. The stress–strain, displacement changes, and some important mechanical parameters of the grass carp skin material during the entire deformation process were analyzed by the binocular stereo DIC measurement system combined with the tensile testing machine. At the same time, the inner and outer layers of fish skin were analyzed from the speckle point of view, and the speckle pattern that was most favorable for DIC matching calculation was selected. The grass carp skin specimen was modeled using the simulation method, and the finite element method (FEM) results verified the global stress–strain measurement accuracy of DIC. The calibrated tensile machine had higher precision than the traditional tensile test, and the full-field high-precision measurement characteristics of DIC were more conducive to the testing of material mechanical properties.

## 2. Measurement Principle of the Binocular Stereo DIC System

### 2.1. Principle of DIC

**Figure 1:**
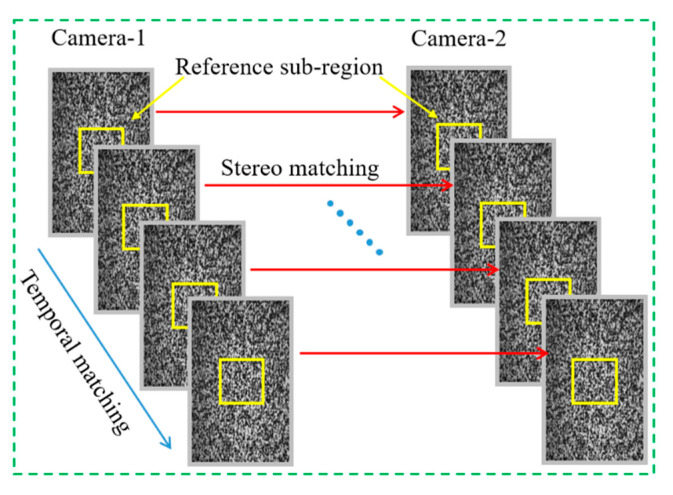
Matching principal diagram of DIC.

The DIC matching process includes stereo and temporal matching. The matching process is shown in Figure 1. First, a rectangular region in the reference image (usually the first image) is selected as a reference subregion, and stereo matching is performed with the corresponding subregion in the deformed image. Second, the speckle images are captured by the same camera are matched in time sequence. The matching process is accomplished by tracking the variation in the speckle points, and the quality of the matching is measured by the mutual coefficient. After the threshold is set, the correlation coefficient is continuously optimized. Based on the study of correlation coefficients by Pan [19], the zero-mean normalized cross-correlation coefficient (*C_ZNCC_*) is selected as follows:

(1) $$C_{ZNCC}=\frac{\sum_{x=-M}^{M}\sum_{y=-M}^{M}\left[f\left(x,y\right)-\bar{f}\right]\cdot \left[g\left(x^{\prime },y^{\prime }\right)-\bar{g}\right]}{\sqrt{\sum_{x=-M}^{M}\sum_{y=-M}^{M}{\left[f\left(x,y\right)-\bar{f}\right]}^{2}}\sqrt{\sum_{x=-M}^{M}\sum_{y=-M}^{M}{\left[g\left(x^{\prime },y^{\prime }\right)-\bar{g}\right]}^{2}}}$$

(2) $$\bar{f}=\frac{1}{{\left(2M+1\right)}^{2}}\cdot \sum_{x=-M}^{M}\sum_{y=-M}^{M}f\left(x,y\right),\;\bar{g}=\frac{1}{{\left(2M+1\right)}^{2}}\cdot \sum_{x=-M}^{M}\sum_{y=-M}^{M}g\left(x,y\right)$$

where $\bar{f}$ and $\bar{g}$ are the average gray value of the reference subregion and the subregion in the deformed image, respectively.

### 2.2. Binocular Imaging

**Figure 2:**
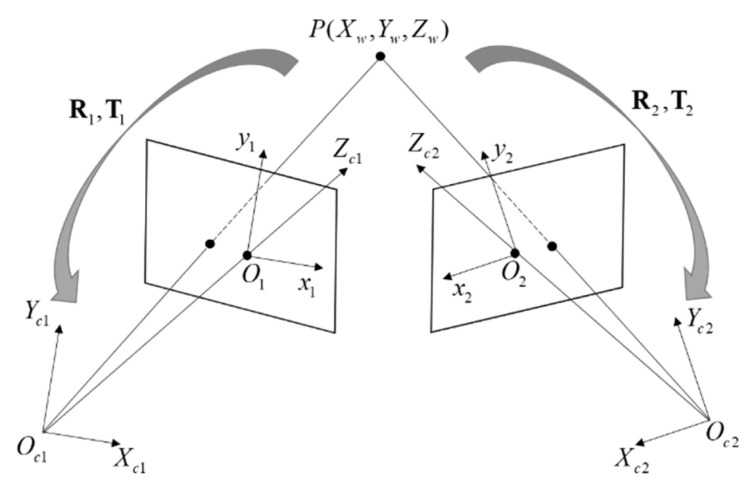
Binocular system imaging model.

In this study, a pinhole imaging model [20,21] was used to solve the 3D coordinates of the object surface, and the imaging model is shown in Figure 2. Zhang’s calibration method [22] was utilized to complete the calibration of the two cameras. Imaging can also be understood as a 2D and 3D coordinate transformation process. Any point *P_w_*(*X_w_*_,_ *Y_w_*_,_ *Z_w_*) on the object surface in 3D space was transformed into a point *P_c_*(*X_c_*_,_ *Y_c_*_,_ *Z_c_*) in the camera coordinates by rigid body. The process only involved the proportional conversion between 3D coordinates, and no change of form was required to obtain the external parameters of the cameras. After perspective projection, the camera coordinates were projected as a point *P*(*X*_,_ *Y*_,_ *Z*) in the image coordinate system. The projection process involved only the internal parameters of the camera. In general, the internal parameters of the camera were a constant value, which could be found once.

The whole transformation process for further clear description can be derived by the formula, and the rigid body transformation is described as

(3) $$P_{ci}=R_{i}P_{w}+T_{i}$$

where *i* = 1,2 correspond to the indices of the left and right cameras, respectively. ***R****_i_* and ***T****_i_* are the rotation matrix and translation vector of the two cameras with respect to the world coordinate system on the calibration plate, respectively. The 2D coordinates could be converted between different units, and the imaging plane coordinates were converted to the form expressed in pixels by the internal parameters of the camera. Therefore, the perspective projection can be written as

(4) $$s\tilde{m}=A[\begin{array}{cc} R & t \end{array}]\tilde{M}$$

Camera internal parameters:$A_{i}=\left[\begin{array}{ccc} f & 0 & c_{x}\\ 0 & f & c_{y}\\ 0 & 0 & 1 \end{array}\right]$.

$\tilde{m}={\left[u,v,1\right]}^{T}$ denotes the expanded coordinates in the form of pixel coordinates, *s* is the scale factor, and $\tilde{M}={\left[X_{w},Y_{w},Z_{w},1\right]}^{T}$ is the expanded coordinates of the world coordinates *P_w_*. Using the coordinates of camera 1 as the reference coordinates of the binocular system, the relationship between the two cameras is

(5) $$\{\begin{array}{c} R=R_{1}\cdot R_{2}\cdot T\\ T=T_{1}-RT_{2} \end{array}$$

The internal and external parameters ***A****_i_*, ***R****_i_* and ***T****_i_* of the two cameras were found by calibration; then, the correspondence between the two binoculars was established.

To eliminate the image distortion caused by the defects of the image acquisition equipment during the imaging process, this study used the radial distortion and tangential distortion models to correct the acquired original images. The distortion correction models can be expressed as follows, respectively.

(6) $$\{\begin{array}{c} x_{distorted}=x\left(1+k_{1}r^{2}+k_{2}r^{4}+k_{3}r^{6}\right)\\ y_{distorted}=y\left(1+k_{1}r^{2}+k_{2}r^{4}+k_{3}r^{6}\right) \end{array}$$

(7) $$\{\begin{array}{c} x_{distorted}=x+2p_{1}xy+p_{2}\left(r^{2}+2x^{2}\right)\\ y_{distorted}=y+2p_{2}xy+p_{1}\left(r^{2}+2y^{2}\right) \end{array}$$

where (*x*, *y*) is the corrected image point,$\left(x_{distorted},y_{distorted}\right)$ is the distorted image point; *k*_1_, *k*_2_, and *k*_3_ are the radial distortion coefficients; *p*_1_ and *p*_2_ are the tangential distortion coefficients; and $r^{2}=x^{2}+y^{2}$.

### 2.3. Speckle Pattern Evaluation Method

Speckle spot, as an indicator of DIC measurement, can reflect the deformation information of the measured object. Thus, the quality of the speckle image affects the accuracy of DIC measurement. The epidermis and dermis of fish skin have different texture structures. The quality of the speckle spot of the samples needs to be evaluated to obtain accurate measurement results. Speckle size, speckle duty ratio, and average grayscale gradient are commonly used speckle quality evaluation indicators [23,24,25]. According to these indicators, high-quality speckle specimens are selected to reduce the effect of speckle on measurement results. The mean gray gradient is defined as follows:

(8) $$\delta_{f}=\frac{\sum_{i=1}^{W}\sum_{j=1}^{H}\left|\nabla f\left(x_{i},y_{j}\right)\right|}{W\times H}$$

where *W* and *H* are the width and height of the speckle image, respectively; $\left|\nabla f\left(x_{i},y_{j}\right)\right|=\sqrt{f_{x}{\left(x_{i},y_{j}\right)}^{2}+f_{y}{\left(x_{i},y_{j}\right)}^{2}}$ is the modulus value of the gray gradient vector of each pixel point.

## 3. Experiments

In this section, the uniaxial tensile experiment of GFSA was implemented, and the tensile testing machine was calibrated using a dual-frequency laser interferometer in advance to improve the accuracy of displacement of the tensile testing machine during the tensile process. Binocular stereo DIC system recorded and analyzed the entire deformation process of GFSA. The displacement and strain accuracy of the method in the GFSA deformation experiments were verified by the displacement data of the tensile machine and the FEM, respectively.

### 3.1. Binocular Stereo DIC Experimental Setup

**Figure 3:**
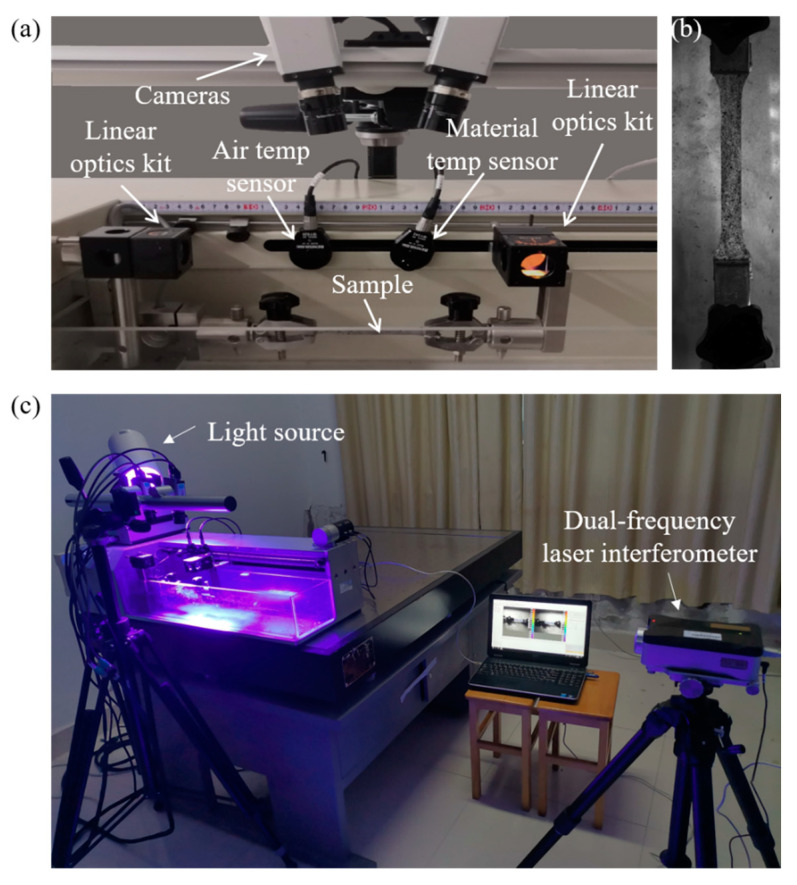
Experimental setup: (**a**) measurement setup; (**b**) GFSA; (**c**) overall setup.

Fresh grass carp skin is a deformable material and has incompressible properties, and obvious wrinkle deformation will occur in the transverse and longitudinal directions during uniaxial stretching. Therefore, other types of deformation experiments are not required to analyze the mechanical properties of grass carp skin materials. The overall experimental setup is shown in Figure 3. A high-precision industrial camera from Germany AVT (resolution: 1624 × 1234 pixels) and two lens with a focal length of 25 mm were used to capture speckle images during the entire deformation process of GFSA. The camera was warmed up for 2 h before the experiment to reduce the thermal error caused by the self-heating of the camera [26]. The blue light illumination system was used to provide stable illumination intensity, and the stable illumination system helps reduce the influence of external light changes on image acquisition. At the same time, the blue light was also beneficial to reducing the interference to the red laser emitted by the dual-frequency laser interferometer (XL-80 produced by RENISHAW).

The uniaxial tensile stress was provided by the tensile testing machine (HVC-WWX) with a force value resolution of 0.01 N and a displacement resolution of 0.01 mm. The left holding head was fixed, and the right holding head was moved to the right at a constant speed through the horizontal lever, setting the stretching speed to 5 mm/min and the strain rate to 0.00167 s^−1^. The clamping head of the tension machine is shown in Figure 3a. The tension machine provided a stable output of uniaxial stretching power during the stretching process.

### 3.2. Accuracy Calibration of Tensile Machine

**Figure 4:**
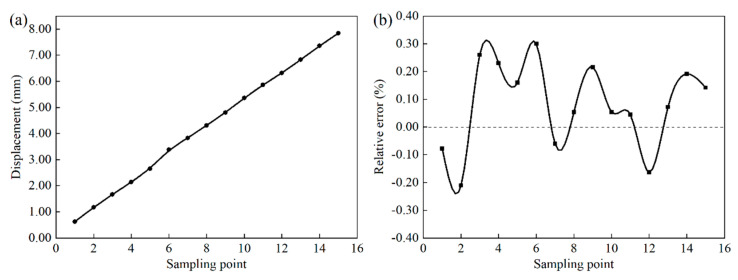
Displacement calibration: (**a**) displacement calibration curve of tensile machine; (**b**) displacement calibration error.

For high-precision measurement, the displacement accuracy of 0.01 mm of the tensile machine could not meet the needs of high-precision measurement. Therefore, the displacement of the tensile machine needed to be calibrated by a dual-frequency laser interferometer before the formal tensile experiment. The specific calibration process was as follows:

The 15 displacement points were read out from the GUI of the tensile machine by manipulating the control button, while the displacement values of the corresponding points were collected using the dual-frequency laser interferometer. The displacement accuracy of the tensile machine was calibrated based on the sampling data of the dual-frequency laser interferometer. After the displacement sampling points are calibrated, as shown in Figure 4a, the sampling curve changed approximately linearly. Meanwhile, Figure 4b shows that the relative error of the displacement between the dual-frequency laser interferometer and the tension machine was between −0.21% and 0.3%. The dual-frequency laser interferometer achieved a good calibration of the tension machine. The displacement accuracy of the tension machine after calibration meets the requirements of high-precision experiments.

### 3.3. Sample Preparation

**Figure 5:**
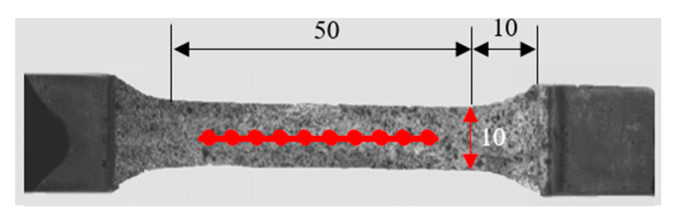
Dimensions of GFSA. (Unit: mm).

In this study, the skin of grass carp was selected as the experimental object. Two kinds of grass carp of the same age and different weight were selected: the first one was 2.5 kg (length 590 mm, width 113 mm); the second one was 1.1 kg (length 430 mm, width 90 mm). The skin of the head, middle, and tail were cut longitudinally. The size of GFSA is shown in Figure 5. The thicknesses of the samples in hydrophilic and dry states were 0.55 mm and 0.4 mm, respectively. The width of the sample was slightly narrower than that of the chuck of the tensile machine from Figure 3b, and the effective field of view of the camera was 100 × 20 mm^2^. The laboratory temperature was kept constant at about 23 °C, and GFSA was divided into two states of hydrophilic and dry for multiple experiments to find its mechanical properties. The skin extraction process did not destroy its surface structure to ensure the integrity of the fish skin. Fresh fish skins were kept in physiological saline for a short period of time without chemical reagents for treatment, and the experiments were completed within 3 days. Meanwhile, the dry state of GFSA was naturally dried at room temperature, and the specimens were covered with a thin plate for pressing to prevent the curling of fish skin during the drying process. The reason is that this condition affects the measurement effect.

**Figure 6:**
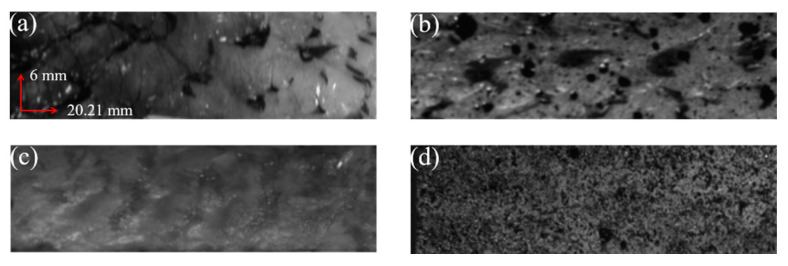
Grayscale images of GFSA: (**a**,**b**) are the effects of the epidermis without artificial speckle and sprayed with artificial speckle, respectively; (**c**,**d**) are the effects of the dermis without artificial speckle and sprayed with artificial speckle, respectively.

Fish skin mainly consists of two layers: epidermis and dermis. The epidermis of grass carp has a black cuticle structure because of its unique skin structure. This structure can easily cause uneven distribution of surface texture. Meanwhile, the inner dermis has a smooth texture. For fish skin soaked in physiological saline, it is necessary to use absorbent paper to absorb the surface moisture before spraying the speckle. As the hydrophilic fish skin itself has a certain toughness, in order to reduce the impact of speckles on the mechanical properties of the fish skin, it is only necessary to spray black paint on the fish skin, so that the surface of the fish skin can form black spots with moderate size and uniform distribution. As shown in Figure 6, Figure 6a,b are the grayscale images of the stratum corneum without artificial speckles and sprayed with artificial speckles, respectively, and Figure 6c,d show the grayscale images of the dermis without artificial speckles and sprayed with artificial speckles, respectively. From the size and uniformity of the speckle, the speckle image in Figure 6d had the best quality.

### 3.4. Speckle Evaluation

**Figure 7:**
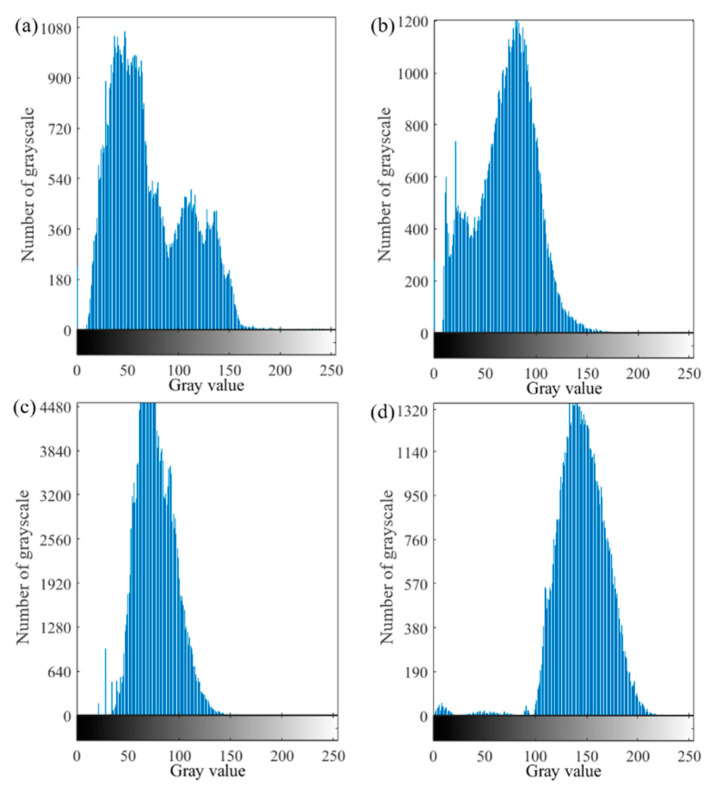
Grayscale histogram of GFSA. (**a**) epidermis without artificial speckle; (**b**) epidermis with artificial speckle; (**c**)dermis without artificial speckle; (**d**)dermis with artificial speckle.

For a good speckle image, the speckle size was between 3 and 5 pixels, the speckle distribution was uniform, the speckle duty cycle was 50%, and the average grayscale gradient was large. The properties of GHSA were judged intuitively as shown in Figure 6. According to the evaluation index of the speckle pattern, the four speckle patterns in Figure 6 were evaluated from the angle of the speckle size, the duty ratio of the speckle, and the average grayscale gradient. The indicators of the four speckle patterns are shown in Table 1. From the three indicators in Table 1, Figure 6d satisfies the characteristics of a good speckle pattern. At the same time, according to the grayscale distribution of the speckle map, the grayscale histograms shown in Figure 7 correspond to the four speckle images in Figure 6. The grayscale values of Figure 7d were mainly distributed between 100 and 200. The brightness of the speckle image was suitable. No noticeable under- or over-exposed speckle grain was observed. The distribution of the grayscale histogram also indicated that the speckle effect shown in Figure 6d was better.

According to the speckle effects in Figure 6 and Figure 7, it was revealed that the grayscale images of the epidermis without artificial speckles and sprayed with artificial speckles exhibited an uneven distribution of speckles in the corneal layer. Since the basis for matching was grayscale information, the larger speckle containing the same grayscale value had a direct effect on the matching result. The grayscale image without artificial speckles in the dermis layer had no obvious speckle features, and the small number of natural speckles was also not conducive to DIC matching.

## 4. Results

The dynamic clamping head was driven by the tensile testing machine to provide the initial loading speed, and the binocular stereo DIC system captured the entire deformation process of the GFSA at a frequency of 0.5 Hz. The GFSA was directly installed on the clamping head. The tensile machine was started after the sample was loaded to give the sample a small displacement for keeping the sample horizontal to avoid wrinkles. This method avoided measurement errors caused by sample twisting or torsion. Then, the two cameras and the tensile testing machine were started synchronously. The experimental setup is shown in Figure 3a.

**Figure 8:**
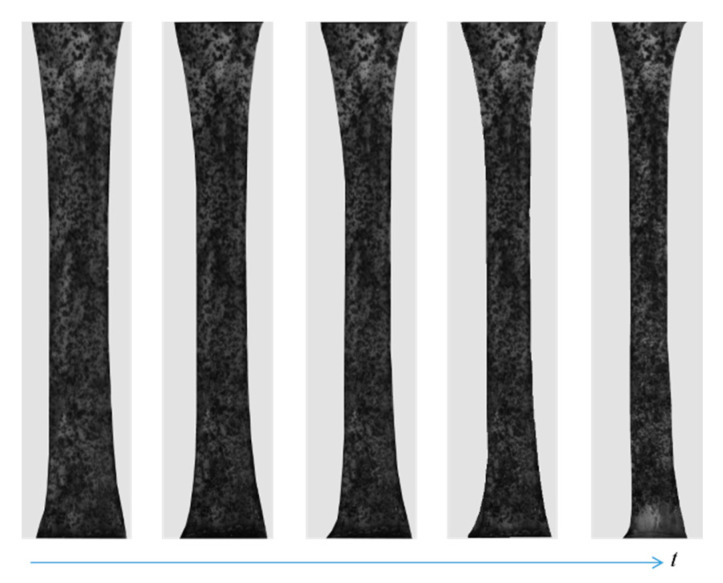
Variation of the specimen.

The deformation of the GFSA in the range of elastic change during tension is shown in Figure 8.

In this study, the mechanical properties of GFSA were considered from the perspective of elastic modulus (*E*) and stress–strain, and *E* was solved using the following formula:

(9) E=FAΔll

where *F* is the axial tensile force; *A* is the cross-sectional area of the sample; Δl and *l* are the displacement elongation and the initial length of the sample, respectively.

**Figure 9:**
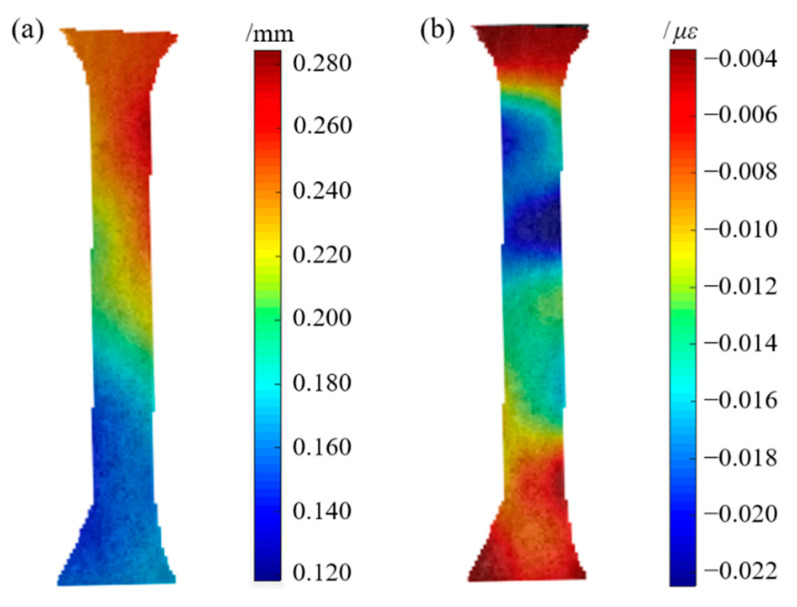
Cloud map of displacement and strain measured by DIC: (**a**) axial displacement; (**b**) axial strain.

**Figure 10:**
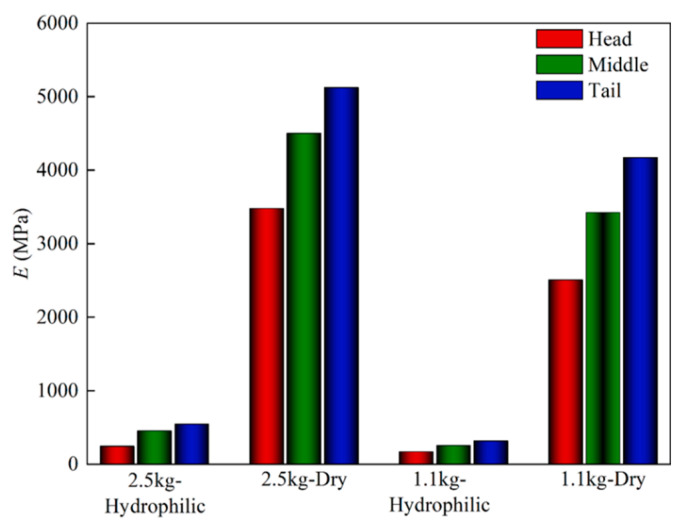
*E* of GFSA.

The full-field axial displacement and strain results of GFSA measured by the binocular stereo DIC system in the elastic range are shown in Figure 9. The specimen was greatly changed during the uniaxial tensile process. The cloud image in Figure 9a shows that the overall change of GFSA was uniform. The axial strains were between 0.004 and 0.022με, and the overall strain changed uniformly. The cloud diagram in Figure 9 shows that DIC can completely calculate the displacement and strain of GFSA. Figure 10 indicates that the fish skin exhibited different *E* values in different states. *E* was relatively small in the hydrophilic state, and *E* showed an opposite trend when the texture of the dried sample became hard. At the same time, the *E* of the head skin was the smallest, and the *E* of the tail skin was the largest; the properties of the middle skin were between those of the head and the tail. Fish skins in different states showed obvious mechanical differences. Differences in mechanical properties also differed among individuals of different maturity levels. In the same individual, *E* had a maximum difference of 2.2 times. Grass carp with different body weights had different maturity levels and skin thicknesses. At the same time, the skin thicknesses of the same parts were also different under different dryness. These factors can lead to controllable errors in the mechanical property values.

Fish swim by tail fin swing. Thus, the tissue structure of the tail skin was more compact, the tissue structure was not easily deformed, and the *E* was relatively larger. Conversely, the skin of the head had the smallest *E*. For the same area of skin; the *E* of adult individuals was greater. Furthermore, the *E* of the dried samples changed drastically.

**Figure 11:**
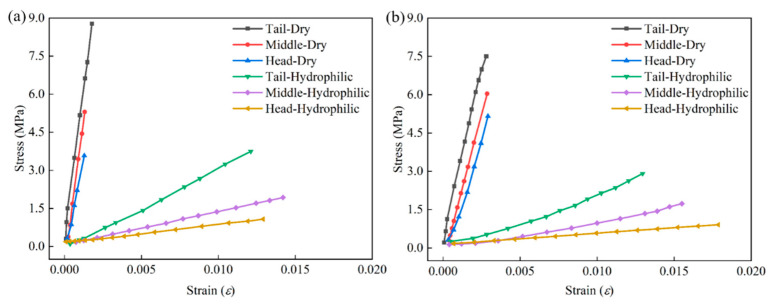
Engineering stress–strain curves of GFSA: (**a**) 2.5 kg; (**b**) 1.1 kg.

**Figure 12:**
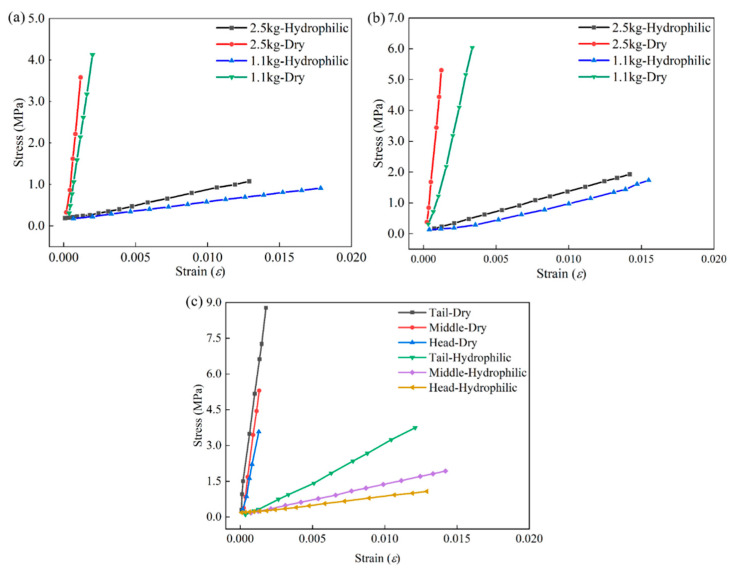
Engineering stress–strain in different parts: (**a**) head; (**b**) middle; (**c**) tail.

Figure 11 and Figure 12 analyze the characteristics of GFSA from the perspective of engineering stress–strain. Fish skin is rich in collagen components. The collagen tissue of the dried GFSA was destroyed, the plasticity was significantly reduced, the strength was increased, and it was more difficult to deform. The engineering stress–strain curve of the dried GFSA during uniaxial stretching was steeper, and the deformation amount decreased under the same stress. The maximum difference in stress was 13 times and the minimum difference was 5 times under the premise of the same strain in the same part of the 2.5 kg individual. A difference of 11 times the maximum stress and 5 times the minimum stress was also observed in the 1.1 kg individual.

Figure 12 shows that the deformation ability of the adult individual fish skin was weaker, and the same strain required a larger tensile strength. The engineering stress–strain trend diagrams of the three parts show that the tensile strength of the dried GFSA was higher than that of the hydrophilic GFSA. From the different mechanical properties of grass carp skin, grass carp skin has great research value.

## 5. Discussion

In this section, comparative experiments are used to verify the accuracy of the proposed method in the measurement of mechanical properties of fish skins. A dual-frequency laser interferometer has high precision and synchronous recording. It was used to verify the displacement accuracy of DIC in GFSA measurement. The strain accuracy of DIC was verified by FEM.

### 5.1. Verification of Displacement Accuracy

**Figure 13:**
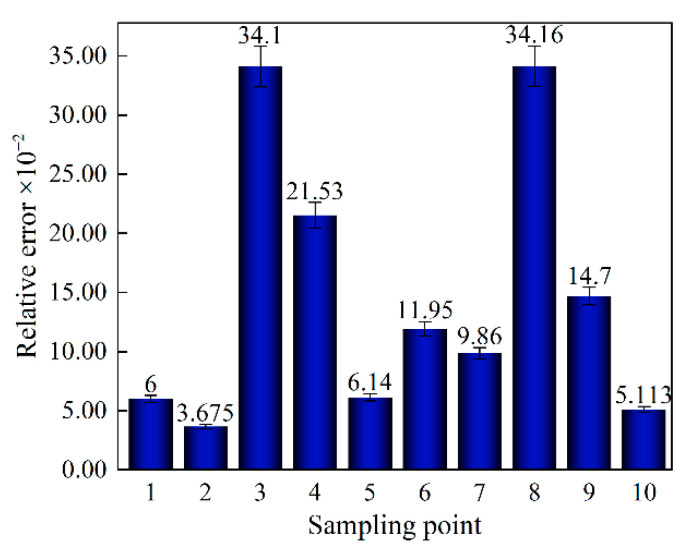
Accuracy of selected sample points.

A dual-frequency laser interferometer can realize the feature of recording displacement synchronously with the tensile machine. Therefore, displacement synchronization with the binocular stereo DIC system was indirectly achieved. Ten calculation points were selected from the middle of the dry-processed samples, as shown in the selected points in Figure 6. The displacement measurement accuracy of the DIC was quantified using the absolute value of the relative error. As shown in Figure 13, the maximum error accuracy of DIC was about 0.342%, which meets the needs of precision measurement. Some inevitable uncertain errors in the experimental process can affect the calculation accuracy. The errors that affect the calculation accuracy mainly include on the one hand, the sample making process. GFSA is a soft biological tissue that is easily deformed, and ensuring that the sample size is completely consistent is difficult. On the other hand, the laser beam of the dual-frequency laser interferometer is disturbed by the external environment.

### 5.2. Verification of Strain Accuracy

Stress–strain is the most intuitive mechanical quantity to express the deformation characteristics of materials. The FEM is also one of the commonly used methods in the global analysis of the mechanical properties of materials. The model structure is reasonably established according to a few mechanical parameters, which can effectively analyze the global properties of the material. FEM is important for selecting a reasonable model in biomaterial analysis, and a good model can ideally represent the mechanical properties of materials. Since fish skin is a material that deforms easily, the nonlinear hyperelastic model does not model the full field well for large deformation cases. Therefore, the strain accuracy of DIC by investigating the single-point strain was verified in this study. According to the modeling scheme proposed in the related literature on mouse skin samples [27], the software of Abaqus was used to model the fish skin material, and the nonlinear hyperelastic Ogden material model was chosen, the mesh type was selected as Free, and the global mesh size was set to 1. The Ogden model is represented as,

(10) $$W=\sum_{i=1}^{N}\frac{\mu_{i}}{\alpha_{i}}\left({{\bar{\lambda }}_{1}}^{\alpha_{i}}+{{\bar{\lambda }}_{2}}^{\alpha_{i}}+{{\bar{\lambda }}_{3}}^{\alpha_{i}}-3\right)+\sum_{i=1}^{N}\frac{1}{D_{i}}{\left(J-1\right)}^{2i}$$

where *N* denotes the order of the model, $\mu_{i}$ and $\alpha_{i}$ are the material constants,$\lambda_{1}$, $\lambda_{1}$ and $\lambda_{1}$ are the principal elongations in the three directions,$J={\left(\lambda_{1},\lambda_{2},\lambda_{3}\right)}^{\frac{1}{2}}$, and *D_i_* is the change in volume.

**Figure 14:**
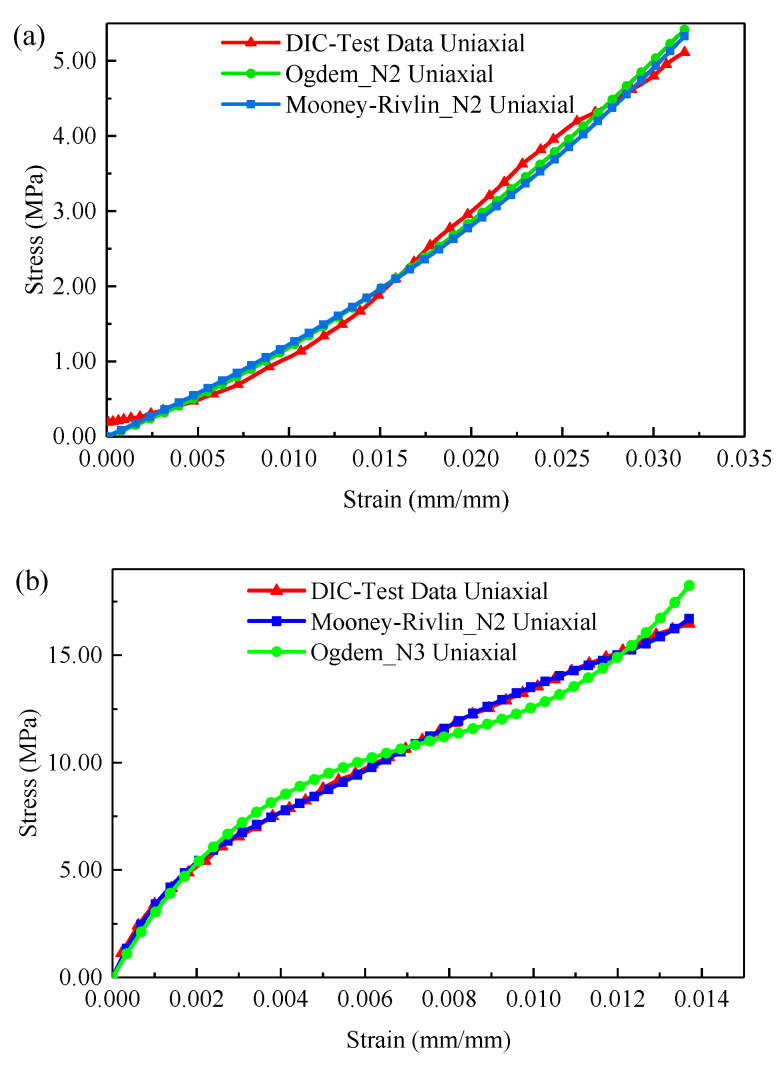
Stress–strain analysis of GFSA: (**a**) 1.1 kg hydrophilic tail; (**b**) 1.1 kg dry tail.

Another group of adult individuals (1.1 kg) were selected for experimental analysis to demonstrate the reliability of the verification results. The calculation results of DIC were compared with those of FEM from the perspective of stress–strain, and the Mooney–Rivlin material model was used as a comparison. The results of the FEM are shown in Figure 14. The stress–strain curves of the three methods had a good fitting effect as shown in Figure 14, and the change trends were relatively consistent. The strain measurement accuracy of the binocular stereo DIC system also met the strain requirements of fish skin materials well.

## 6. Conclusions

In this study, a novel measurement method was proposed to study the mechanical properties of fish skin materials. The binocular stereo DIC system was well-applied to the analysis of the mechanical properties of soft tissues such as fish skin with the aid of peripherals. The uniaxial tensile experiments of grass carp skins of two different individuals showed that the skins of the head, middle, and tail had obvious differences in mechanical properties, and the *E* of the head skin was the smallest. The properties of the tail skin were opposite those of the head skin; meanwhile, the *E* of the middle skin was between those of the head and tail. A maximum difference of 2.2 times was observed between the *E* of the head and tail skin of the same individual. The tensile strength of the dried GFSA was higher than that of the hydrophilic GFSA. Under the same stress, the amount of strain at the tail was the smallest. The dermal layer of grass carp skin had a smooth structure and uniform texture, and the artificial spraying speckle pattern was more suitable for the speckle requirements of binocular stereo DIC. The dual-frequency laser interferometer verified the high displacement accuracy of the binocular stereo DIC system, and the high strain measurement capability of the system was proven using FEM.

The application of DIC in fish skin materials also provided a new method for the study of mechanical properties of other biological soft tissues. The study of grass carp skin in the hydrophilic state can provide data support and research methods for the preparation of flexible materials. The dried grass carp has a hard texture and is not easily deformed. It also has potential application value in leather and composite materials.

## Figures and Tables

**Table 1:** Evaluation index of the speckle pattern.

Sequence	Size/Pixel	Duty Cycle	Average Grayscale Gradient
a	18.78	65.95%	3.87
b	13.12	37.19%	6.05
c	25.08	54.87%	2.80
d	4.91	55.64%	9.08

## Data Availability

Not applicable.

## References

[1] Lee G.J., Nam W.I., Song Y.M. (2017). Robustness of an artificially tailored fisheye imaging system with a curvilinear image surface. Opt. Laser Technol..

[2] Dardari D., Lequint C., Jugnet A.C., Benard T., Bouly M., Penfornis A. (2022). Curing Necrotic Angiodermatitis with an Intact Fish Skin Graft in a Patient Living with Diabetes. Medicina.

[3] Szewciw L., Barthelat F. (2017). Mechanical properties of striped bass fish skin: Evidence of an exotendon function of the stratum compactum. J. Mech. Behav. Biomed. Mater..

[4] Seo E., Park J., Gil J.-E., Lim H., Lee D., Lee S.J. (2021). Multifunctional biopolymer coatings inspired by loach skin. Progress Org. Coat..

[5] Bian C., Huang Y., Li J., You X., Yi Y., Ge W., Shi Q. (2019). Divergence, evolution and adaptation in ray-finned fish genomes. Sci. China Life Sci..

[6] Vernerey F.J., Barthelat F. (2014). Skin and scales of teleost fish: Simple structure but high performance and multiple functions. J. Mech. Phys. Solids.

[7] Jo W., Kang H.S., Choi J., Jung J., Hyun J., Kwon J., Kim I., Lee H., Kim H.-T. (2021). Light-Designed Shark Skin-Mimetic Surfaces. Nano Lett..

[8] Acciaioli A., Falco L., Baleani M. (2020). Measurement of apparent mechanical properties of trabecular bone tissue: Accuracy and limitation of digital image correlation technique. J. Mech. Behav. Biomed. Mater..

[9] Chen B., Pan B. (2021). Measuring true stress–strain curves of cylindrical bar samples with mirror-assisted multi-view digital image correlation. Strain.

[10] Attia T., Di Benedetto H., Sauzéat C., Pouget S. (2021). Behaviour of an interface between pavement layers obtained using Digital Image Correlation. Mater. Struct..

[11] Mohamadizadeh A., Biro E., Worswick M. (2021). Novel Double-Half Spot Weld Testing Technique For Damage Progress And Failure Analysis Using Digital Image Correlation Techniques. Exp. Mech..

[12] Baldi A. (2020). Robust Algorithms for Digital Image Correlation in the Presence of Displacement Discontinuities. Opt. Lasers Eng..

[13] Bao S., Wang Y., Liu L., Lu Y., Yan P. (2019). An error elimination method for high-temperature digital image correlation using color speckle and camera. Opt. Lasers Eng..

[14] Yz A., Lf B., Sd C., Jl A. (2022). Full-field deformation measurements in the transmission electron microscope using digital image correlation and particle tracking. Mater. Charact..

[15] Palanca M., Tozzi G., Cristofolini L. (2015). The use of digital image correlation in the biomechanical area: A review. Int. Biomech..

[16] Morosato F., Traina F., Cristofolini L. (2019). A reliable in vitro approach to assess the stability of acetabular implants using digital image correlation. Strain.

[17] Chen Y.F., Dang N.M., Wang Z.Y., Chang L.W., Ku W.Y., Lo Y.L., Lin M.T. (2021). Use of Digital Image Correlation Method to Measure Bio-Tissue Deformation. Coatings.

[18] Cisar P., Bekkozhayeva D., Movchan O., Saberioon M., Schraml R. (2021). Computer vision based individual fish identification using skin dot pattern. Sci. Rep..

[19] Pan B., Xie H., Wang Z. (2010). Equivalence of digital image correlation criteria for pattern matching. Appl. Opt..

[20] Zhu C., Shao X., Liu C., He X. (2019). Accuracy analysis of an orthogonally arranged four-camera 3D digital image correlation system. Appl. Opt..

[21] Feng M., Huang S., Wang J., Yang B., Zheng T. (2017). Accurate calibration of a multi-camera system based on flat refractive geometry. Appl. Opt..

[22] Zhang Z. (2000). A flexible new technique for camera calibration. IEEE T. Pattern Anal..

[23] Dong Y.L., Pan B. (2017). A Review of Speckle Pattern Fabrication and Assessment for Digital Image Correlation. Exp. Mech..

[24] Su Y., Gao Z., Fang Z., Liu Y., Wang Y., Zhang Q., Wu S. (2019). Theoretical analysis on performance of digital speckle pattern: Uniqueness, accuracy, precision, and spatial resolution. Opt. Express.

[25] Su Y., Zhang Q. (2022). Glare: A free and open-source software for generation and assessment of digital speckle pattern. Opt. Lasers Eng..

[26] Pan B. (2018). Thermal error analysis and compensation for digital image/volume correlation. Opt. Lasers Eng..

[27] Karimi A., Navidbakhsh M., Haghighatnama M., Haghi A.M. (2015). Determination of the axial and circumferential mechanical properties of the skin tissue using experimental testing and constitutive modeling. Comput. Methods Biomech. Biomed. Eng..

